# Microfluidic paper-based device coupled with 3D printed imaging box for colorimetric detection in resource-limited settings

**DOI:** 10.1016/j.ohx.2023.e00456

**Published:** 2023-07-14

**Authors:** Vijay Vaishampayan, Oinam Robita Chanu, Balasubramanian Sivasamy, Muthamilselvi Ponnuchamy, Varshini Karthik, Ambar Pendharkar, Lohith Srinivas Thotakura, Aryan Prabhu, Venkatesan Dhananjeyan, Ashish Kapoor

**Affiliations:** a Department of Chemical Engineering, Indian Institute of Technology, Ropar, Rupnagar, Punjab 140001, India; b Department of Biomedical Engineering, SRM Institute of Science and Technology, Kattankulathur, Tamil Nadu 603203, India; c Department of Chemical Engineering, KPR Institute of Engineering and Technology, Coimbatore, Tamil Nadu 641407, India; d Department of Chemical Engineering, SRM Institute of Science and Technology, Kattankulathur, Tamil Nadu 603203, India; e Department of Chemical Engineering, Sathyabama Institute of Science and Technology, Chennai, Tamil Nadu 600119, India; f Department of Chemical Engineering, Harcourt Butler Technical University, Kanpur, Uttar Pradesh 208002, India

**Keywords:** Paper-based devices, Imaging box, 3D Printing, Colorimetric detection, Sensor

## Abstract

Rapid and effective methods for the detection of analytes such as water contaminants, food adulterants and biomolecules are essential for the protection of public health and environmental protection. Most of the currently established analytical techniques need sophisticated equipment, centralized testing facilities, costly operations, and trained personnel. Such limitations make them inaccessible to the general populace, particularly in regions with limited resources. The emergence of microfluidic devices offers a promising alternative to overcome several such constraints. This work describes a protocol for fabricating a low-cost, open-source paper-based microfluidic device using easily available tools and materials for colorimetric detection of analytes. The ease and simplicity of fabrication allow users to design customized devices. The device is coupled with an imaging box assembled from 3D printed parts to maintain uniform lighting conditions during analytical testing. The platform allows digital imaging using smartphones or cameras to instantaneously capture images of reaction zones on the device for quantitative analysis. The system is demonstrated for detecting hexavalent chromium, a toxic water contaminant. The image analysis is performed using open-source ImageJ for quantification of results. The approach demonstrated in this work can be readily adopted for a wide range of sensing applications.

## Graphical abstract

**Figure:**
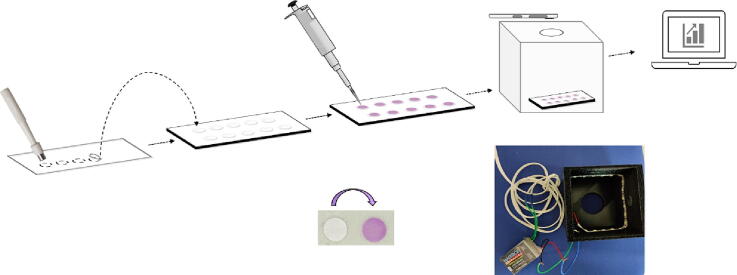

Specifications table

**Table:** 

Hardware name	Paper-based device coupled with imaging box (PD-iBox)
Subject area	Engineering and materials science Environmental, planetary and agricultural sciences Educational tools and open-source alternatives to existing infrastructure Biological applications
Hardware type	Imaging tools Other [Chemical Sensor]
Open source license	Creative Commons Attribution-ShareAlike 4.0 International License (CC BY-SA 4.0)
Cost of hardware	Cost of the paper-based device: 7 INR (Without reagents) Cost of the imaging box: 1600 INR
Source file repository	https://doi.org/10.17632/p7jjtrt82w.4

## 1 Hardware in context

Detection of analytes is of prime importance in environmental monitoring, food quality testing, biomedical diagnostics and industrial operations. Rapid and accurate determination of contaminants and pathogens plays a crucial role in ensuring public well-being and environmental safety [1], [2]. The currently used analytical methods involve usage of sophisticated techniques such as atomic absorbance spectroscopy, inductively coupled mass spectrometry and ion chromatography [3], [4]. Although such established techniques are highly advanced and accurate, they are expensive and not easily accessible. The conventional analytical approach relies on sample collection from sites and transportation to centralized testing facilities that host the state-of-the-art instruments. Special arrangements need to be made to preserve samples in their original state till testing. The entire procedure is conducted by trained professionals. All these factors substantially add to the overall costs of analytical assay. The aforementioned limitations pose constraints in making the analytical testing within easy reach of the common public for widespread usage, especially in resource-limited settings [5], [6], [7]. Consequently, the regions and countries with constrained resources and less educated populations that need these facilities the most have to suffer. The consumption of contaminated water and lack of proper disease diagnostics in these regions have been the causes of numerous health problems. Thus, there is a need for affordable and user-friendly technologies that can be used by common people for rapid detection of contaminants [8], [9], [10], [11].

Paper-based analytical devices that uses colorimetric reaction have evolved as promising tools for the detection of various desired or undesired constituents in food, water, healthcare, biological, and environmental applications [12], [13]. These devices are also referred as lab-on-paper or microfluidic paper-chip devices [14]. Paper consists of a large number of internally connected cellulose-based microchannels in the form of a capillary network. This network provides pathways for the fluids to transfer from one location to another by virtue of capillary action [15], [16]. Paper as a substrate in analytical devices has several unique advantages such as its abundant availability, ease of fabrication, ability to transport fluids without any external pumping devices, flexibility and portability. Further, the white background of commonly used paper makes it useful for colorimetric analysis [17], [18]. These devices require very small volumes of reagents and samples compared to conventional analytical devices. This significantly reduces costs related to operations and logistics [19]. With increasing digitalization, the use of smartphones has pervaded all aspects of our lives [6], [20]. The integration of paper-based analytical devices with the now ubiquitous smartphone helps to capture the image of color formed on the paper substrate. The digital images can be analyzed to obtain color intensities with the help of open-source software like ImageJ [21], [22], [23]. However, this technique requires controlled lighting conditions to capture reliable image data. Although the colors read by the smartphones are sufficient to understand the presence of target analytes, there is still lack of affordable, low cost, reliable detection device with controlled lighting conditions for the sensing applications [1], [24].

Imaging boxes with various functionalities have been reported in the literature to capture digital photographs of experimental setups. In order to establish a digital imaging system for titrations in microplates, a recycled paper box painted with black matte ink was constructed as an image acquisition chamber [25]. The illumination was provided by white LEDs connected to a LED tape controller. An acrylic plate was used as the light diffuser. A manual door mechanism was used to place the microplate supported on a small fibreboard table inside the box. Although the usage of recycled material made the box environmentally friendly and cost-effective, its durability could be a concern. A photography lightbox using cardboard was designed with black fabric internal lining for imaging microtiter plates [26]. The base formed by sticking a foam layer below a perfboard was used for soldering circuit components, including a planar light source. Even though a more significant backlight could illuminate the entire plate at once, the camera would have to be placed farther away to limit the angular dispersion in optical path lengths across the samples. A 3D printed ultraviolet (UV) imaging box was fabricated from polylactic acid with UV-LEDs for fluorescent imaging of quinine in beverages [27]. The box was also provided with coolers to facilitate heat dissipation. However, the box was explicitly meant for fluorescence imaging. An infrared lightbox system coupled with a no-infrared filter camera was devised for phosphate detection via molybdenum blue reaction protocol [28]. Infrared LEDs in the sophisticated colorimetric analyzer enabled reproducible illumination conditions and provided accurate image acquisition in the infrared spectrum. An acrylic light box was fabricated with a circular opening on one of the vertical sides for imaging with digital still cameras [29]. White LEDs were placed around the hole provided for the camera lens. A sample holder was set at the opposite end to accommodate standard glass cuvettes and test tubes. The arrangement provided a fixed imaging distance and was specifically designed for imaging liquid samples placed in cuvettes and test tubes.

Hence, most reported imaging boxes incorporate high-end mechanical or electronic components for specific applications. Consequently, these features make them costly and often bulky. Further, trained professionals are needed for the upkeep of such equipment. At the other extreme are cheaper imaging boxes that lack sufficient features that limit their usage. The imaging box proposed in this work is simple and sturdy and has versatile features for typical analytical applications in regions with limited resource access. Further, the imaging box can be operated and maintained by users with basic technical knowledge. Finally, we present the integration of the imaging box with paper-based analytical devices. Hence, the overall scope of this work is to design and fabricate a low-cost paper-based analytical device along with a light weight, compact and sturdy 3D printed light box that offers controlled lighting conditions to capture digital colorimetric signals. We also demonstrate the successful application of the platform with smartphone-assisted imaging for detecting hexavalent chromium in water. The combined hardware setup opens up the possibilities of several scientific applications of societal relevance.

## 2 Hardware description

### 2.1 Fabrication of paper-based device

**Fig. 1:**
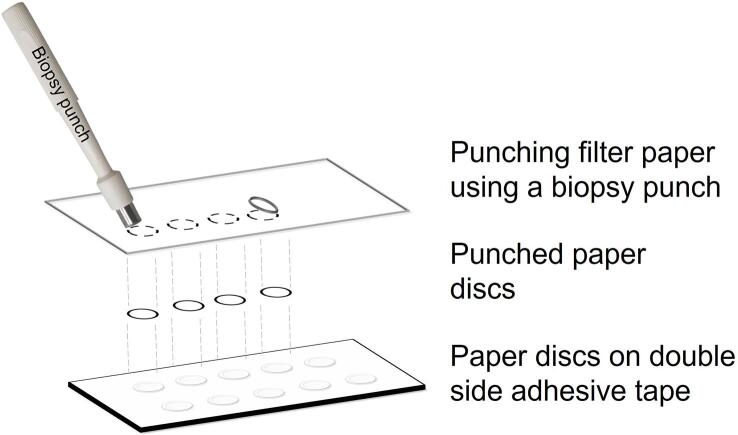
Schematic illustration of fabrication of paper-based device using a biopsy punch.

The Whatman grade 1 filter paper was taken as a substrate for the sensing platform. The circular paper discs of diameter 6 mm were cut using a biopsy punch. These circular discs were adhered at uniform distances on the double-sided adhesive tape. The tape support was 20 mm wide and 2 mm thick. The length of the tape support was 40 mm. It could accommodate five paper discs placed at an edge-to-edge spacing of 2 mm vertically and horizontally, in two parallel rows. The length of the tape can vary depending on the user requirements. The user can place paper discs at different spacing. However, it must be ensured that adjacent paper discs should not touch each other. The tape support not only prevented leakage of fluid underneath but also provided sturdiness to the assembly for ease of handling the device during the operations. The fabricated devices were stored in a clean and dry environment before further use. The steps involved in fabrication are presented in the Fig. 1.

### 2.2 Fabrication of the imaging box

**Fig. 2:**
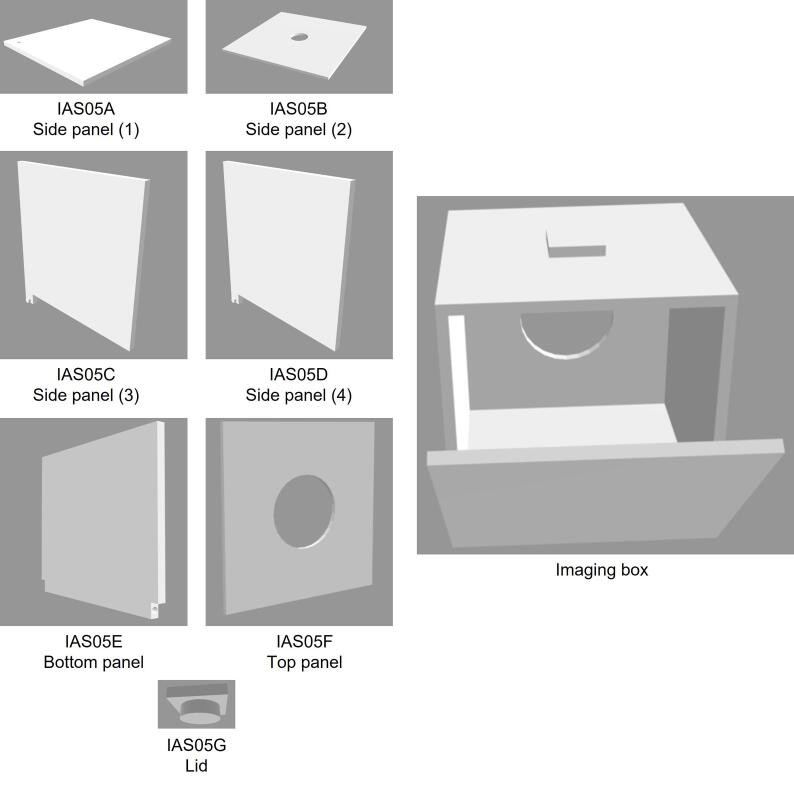
The 3D rendered models for the imaging box (image acquisition system).

3D printing of polymers is advantageous as it allows the printing of cost-effective designs for customized applications. Different complex geometries can be printed in a short period using 3D printing technology, leading to rapid fabrication of prototypes. Various polymeric materials are used for 3D printing, such as acrylonitrile butadiene styrene (ABS), acrylonitrile styrene acrylate (ASA), polylactic acid (PLA), polycarbonate (PC), etc. Here, ABS was used as a fabrication material for the extrusion 3D printing of the imaging box, also called image acquisition system (IAS) or lightbox. In the extrusion 3D printing, the material melted and extruded through the nozzle. The nozzle gave a particular thickness to the material and deposited it in the layers during the fabrication [30], [31], [32], [33]. The imaging box was assembled from 3D printed parts as shown in Fig. 2.

The box was black in color because of use of black colored ABS polymer filament. The overall length, breadth, and height of the box were 12 cm each. The thickness of the top and bottom panels was set at 2 cm each. A circular hole or opening of diameter 5 cm was made in the top panel during the 3D printing process for enabling image acquisition. A provision was also made in one of the side panels to have 2 cm hole for inserting cuvettes and small test tubes for taking images of liquid samples. The panel was referred to as the front panel. The panel opposite to it was designated as the back panel. A lid was designed and provided to plug the circular hole in the front panel when unused. A flap with a hinge mechanism was provided in the model for the easy insertion of the samples. It served as the bottom panel. A LED strip was glued inside the box, providing uniform and focused lighting while capturing the images. The strip comprised of 30 white LEDs having color temperature of 6000 Kelvin. The strip covered all four internal walls of the box and was located at 6 cm height from the bottom panel. The wires of the LED were connected with the power source through a tiny wire insertion hole provided on the back panel. The LEDs were linked with the connector and alternating current to direct current power supply adapter with output parameters of 12 Volts and 5 Amperes. The wire insertion hole was 2 mm in diameter and its center was located at 1.5 cm each from top and side edges on the back panel. A black tape was used as a covering to block the entry of any external light through the hole. The samples to be photographed were placed inside the box on the bottom panel and image acquisition was performed without the interference of external lighting conditions.

### 2.3 Design files

#### 2.3.1 Design files summary

**Table:** 

Design file name	File type	Open source license	Location of the file
side_panel_(1)_IAS05A	.stl	CC BY-SA 4.0	https://doi.org/10.17632/p7jjtrt82w.4
side_panel_(2)_IAS05B	.stl	CC BY-SA 4.0	https://doi.org/10.17632/p7jjtrt82w.4
side_panel_(3)_IAS05C	.stl	CC BY-SA 4.0	https://doi.org/10.17632/p7jjtrt82w.4
side_panel_(4)_IAS05D	.stl	CC BY-SA 4.0	https://doi.org/10.17632/p7jjtrt82w.4
bottom_panel_IAS05E	.stl	CC BY-SA 4.0	https://doi.org/10.17632/p7jjtrt82w.4
top_panel_IAS05F	.stl	CC BY-SA 4.0	https://doi.org/10.17632/p7jjtrt82w.4
lid_S05G	.stl	CC BY-SA 4.0	https://doi.org/10.17632/p7jjtrt82w.4

IAS05 – Image acquisition system 05 A to F.

S05G – Image acquisition system lid.

### 2.4 Bill of materials

#### 2.4.1 Bill of materials summary

**Table:** 

Designator	Components	Number	Cost per unit Indian National Rupees (INR)	Source of materials	Material type
IAS01	ABS Plastic filament	1	985	product link	Polymer
IAS02	Micro LED strip	1	249	product link	Other
IAS03	Insulated wire (0.5 mm)	1	99	product link	Other
IAS04	Power supply adapter with connector (12 V, 5 A)	1	399	product link	Other
LBPCH01	Potassium Dichromate (100 g)	1	230	product link	Inorganic
LBPCH02	Sulfuric Acid (500 mL)	1	3388	product link	Inorganic
LBPCH03	1,5-diphenylcarbazide (25 g)	1	5770	product link	Organic
LBPCH04	Acetone (4000 mL)	1	224	product link	Organic
LBP01	Whatman paper grade 1 (100 sheets)	1	1210	product link	Other
LBP02	Biopsy punch	1	99	product link	Other
LBP03	Double-sided adhesive tape	1	114	product link	Other
LBP04	Micropipettes 1 µL to 10 µL	1	14,376	product link	Other

IAS: Image acquisition system materials ID.

LBPCH: Paper-based device detection chemicals ID.

LBP: Paper-based device fabrication and detection items ID.

Cost calculation of the paper-based device is based on the proposed design.

## 3 Build instructions

### 3.1 Safety instructions

The following safety instructions must be followed during the 3D printing of the imaging box.

• Clean the work surface thoroughly using wet and dry-cleaning methods before starting printing.
• Always use personal protective equipment (PPE) such as aprons, safety gloves, and safety goggles during operations.
• During the printing operations, do not inhale the particulates created when the printing is in progress.
• If the nozzle of the printer is jammed, do not touch and remove it directly. Please turn off the printer and allow it to ventilate before removing it.
• Ensure that the 3D printing must be done in a clean and ventilated place.

### 3.2 Materials of construction

The analytical detection device was constructed using a Whatman grade 1 filter paper. The circular paper discs were punched out using a 6 mm diameter biopsy punch. The paper discs were stuck on one side of the double-sided adhesive tape.

Acrylonitrile butadiene styrene (ABS) was used to fabricate the imaging box by 3D printing. ABS is an opaque thermoplastic material used mainly for fused deposition modeling (FDM) and fused filament fabrication (FFF) techniques in 3D printers [34], [35]. During the printing process, ABS filament is guided into an extrusion head that heats the ABS filament to the required melting point.

### 3.3 Printing configuration

Flashforge Adventurer 3 ultra-mute 3D printer was used for printing the case for the imaging box. The inbuilt creative filament feeding design was used to easily insert the filaments and load them into the nozzles. The maximum permissible heating temperature of the nozzle was 240 °C with a printing speed of 30–100 mm/s. Due to heated lower surface, the objects being printed are attached to the surface tightly. The printer could accommodate the maximum printing volumetric dimensions of 150 mm × 150 mm × 150 mm. User-friendliness, touch screen operations, and ultra-mute printing make 3D printing very convenient for fabricating complex designs.

### 3.4 Multipurpose design

**Fig. 3:**
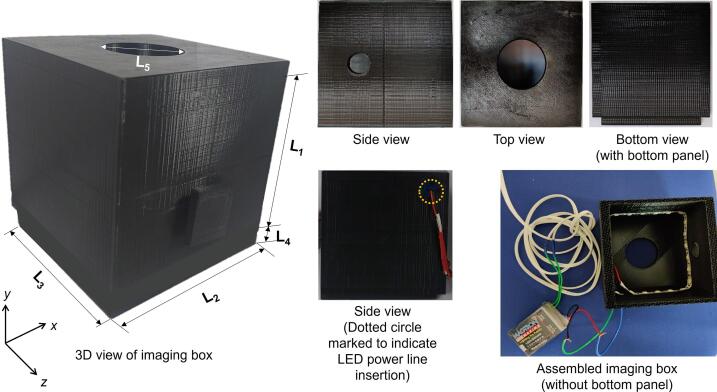
The photographs of the imaging box assembled from 3D printed parts. The dimensions of the imaging box indicated in 3D view are L_1_ = 12 cm, L_2_ = 12 cm, L_3_ = 12 cm, L_4_ = 2 cm, L_5_ = 5 cm.

The designed imaging box provides uniform lighting conditions, which leads to less signal noise and minimal variations in illumination of the object to be imaged. The top panel of the box has an opening for placing the image acquisition device such as a smartphone to acquire the images [36], [37]. The side adjacent to this has an opening of 2 cm, allowing the user to insert a cuvette or test tube for imaging purposes. The fabricated box can aid in image acquisition from paper-based sensors fabricated here as well as from standard sample holding accessories like cuvettes and test tubes used in analytical testing. The system can be used for various analytical and bioanalytical applications [11], [19], [38]. The actual photographs of the imaging box are shown in the Fig. 3 with various views. A 3D view of imaging box showing all dimensions is also presented along with reference coordinate system. A dotted circle is marked on the back panel to indicate power line insertion for connecting the LED with the power source. The proposed hardware set up is simple and affordable for the detection of analytes in resource-limited settings.

## 4 Operation instructions

### 4.1 Procedure to use microfluidic paper-based device

**Fig. 4:**
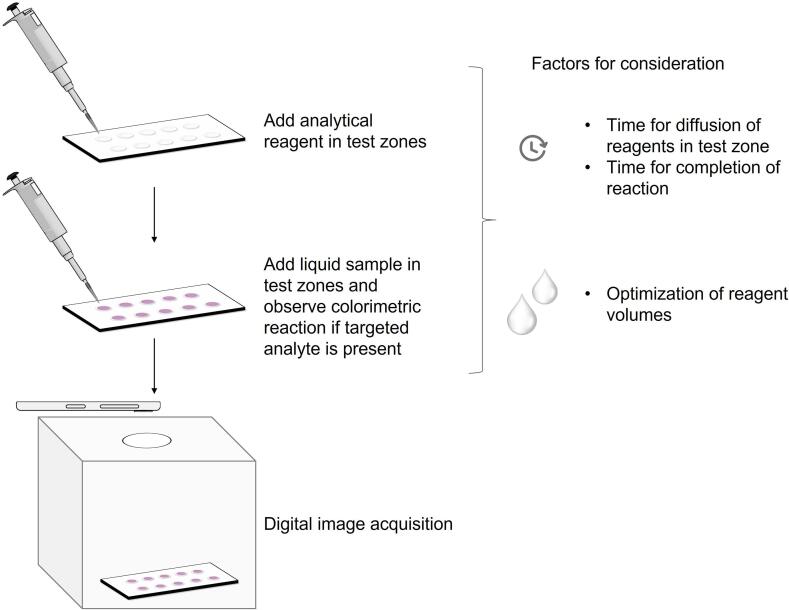
Procedure for performing colorimetric assay on paper-based device and acquiring digital images using imaging box.

The paper-based device is used as a sensing platform to detect the analyte in the aqueous solution. Fig. 4 illustrates the procedure for using paper-based device for colorimetric detection along with image acquisition in the imaging box. The circular paper discs serve as test zones for carrying out the analytical reaction. The optimization of fluid volumes is identified as an essential criterion for the operation of paper-based devices. Preliminary trials should be conducted to determine optimal volume requirements for each test zone. The excess fluid volumes introduced onto the paper disc result in overflow of fluid, whereas low fluid volumes do not cover the entire test zone. The volume ratio of reagent and analyte is also crucial in obtaining the strong intensity optical signal in case of colorimetric reaction. Preliminary experiments should be done considering these factors to determine the actual volume of reagent and analyte that should be used for performing the colorimetric sensing. In order to perform the assay, the pre-determined volume of reagent is introduced onto the test zone and allowed to spread uniformly. Fluid permeates in the paper matrix and flows by capillary action to cover the circular zone. Time required for the coverage depends on fluid transport properties for a particular test zone of fixed dimension. Sufficient time should be allowed to ensure full coverage of the test zone. Subsequently, the pre-determined volume of sample to be tested for the presence of analyte is introduced onto the same test zone. The reaction is allowed to occur between the reagent and the analyte. The presence of the analyte is indicated by the appearance of color in the test zone resulting from the formation of a colored reaction product [17].

### 4.2 Procedure to use imaging box

**Fig. 5:**
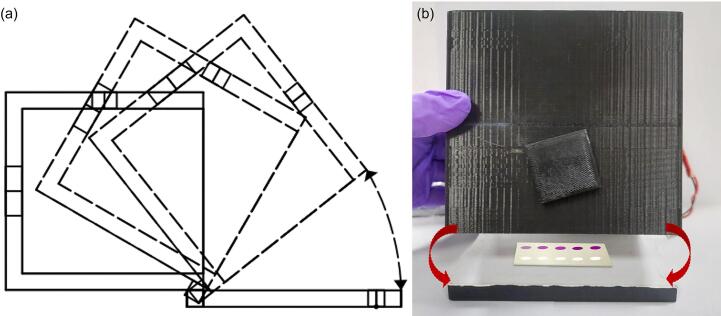
(a) Schematic representation of the side view of the imaging box indicating the mechanism for opening and closing the box, and (b) illustration of placement of the device or sample on the bottom panel by rotating the top portion of the imaging box.

The image acquisition of the paper-based device is made using the 3D printed imaging box before and after performing the colorimetric assay. LED lights should remain switched on during the imaging procedure to ensure uniform lighting conditions. In order to capture image in the pre-assay condition, the blank paper-based device is placed in the box. The hinge mechanism allows the bottom panel and rest of the top portion of the box to rotate with respect to each other. The bottom panel is kept in a horizontal position and the top portion of the box is rotated around the hinge to open the box as shown in Fig. 5(a). The black arrow denotes the direction of rotation of the top portion of the box. The device is placed on the bottom panel and the top portion of the box is rotated back to close the box. The side panel circular hole is kept closed with a lid. The image is acquired using a smartphone placed at the circular opening provided in the top panel. Since all sides are enclosed, no external light enters during image acquisition. After completing the sensing reaction on the paper discs, the device is again placed inside the imaging box and digital image is captured under same settings. The dimensions of the fabricated imaging box are suitable to accommodate commonly available smartphones for image acquisition. The overall setup remains stable unless intentionally or accidentally disturbed. Fig. 5(b) illustrates the placement of paper-based device on the bottom panel by opening and closing the top portion of the box. The red arrows indicate the angular movement of the top portion of the box. The device shown in the figure is placed on the top of a white sheet to prevent any contamination while handling. Same procedure is followed for removing the device after capturing the images. The acquired digital images are used for quantitative analysis of data [6], [11], [39].

### 4.3 Validation and characterization

**Fig. 6:**
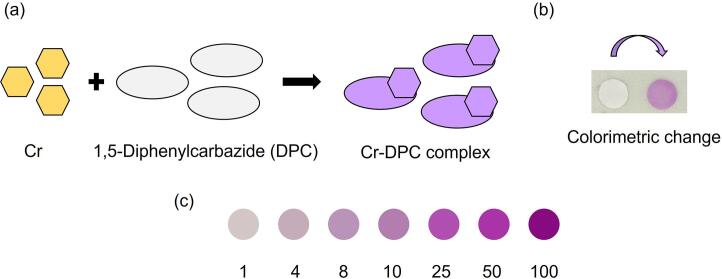
Colorimetric reaction for detection of hexavalent chromium. (a) Schematic representation of reaction illustrating reaction between chromium species and 1,5-Diphenylcarbazide (DPC) to form purple colored Cr-DPC complex, (b) digital photograph for colorimetric reaction performed on paper-based device, and (c) colorimetric intensity for various concentrations of chromium. All the concentration values are in ppm. (For interpretation of the references to color in this figure legend, the reader is referred to the web version of this article.)

The performance of the microfluidic paper-based device coupled with a 3D printed imaging box was validated by examining the colorimetric detection of hexavalent chromium in water samples. The smartphone was used for acquiring digital colorimetric images. The images were analyzed using ImageJ software to obtain color intensity values [40]. The calculations were performed with Microsoft Excel to obtain quantitative relations between intensity values and chromium concentration. Alternatively, any other open-source spreadsheet software may also be used for quantification. The detection of hexavalent chromium is based on its reaction with 1,5-diphenylcarbazide (DPC) [41]. Under acidic conditions, a redox reaction occurs between the reactants and a purple-colored complex appears as shown in Fig. 6(a) [42]. In order to characterize the device, the colorimetric reaction was performed on the circular paper discs designated as test or reaction zones. The amounts of reagents required were determined with the help of preliminary trials. It was ensured that the liquid was sufficient to cover the entire substrate and at the same time did not overflow. 2–10 µL liquid volume range was determined to be optimal for the test zone. Also, the volume ratios of reagents and chromium containing aqueous samples were selected such that the highest color intensity was obtained for a fixed analyte concentration. Accordingly, 2 µL each of 1,5-diphenylcarbazide and sulfuric acid were added to the paper substrate. After the addition, a waiting period of 1 min was given to spread the analyte solution over the whole area of the test zone. Subsequently, 3 μ L of water sample containing chromium was added to the substrate and allowed to react. Again, a waiting period of 1 min was provided before image acquisition to ensure completion of the complex formation reaction on the substrate. The colorimetric changes were digitally captured using a smartphone camera (autofocusing feature,12 MP sensor, aperture: f/1.9, focal length: 4.7 mm, automatic shutter speed with range: 1/1000–32 s, and pixel size: 0.7-µm) with the aid of the 3D printed imaging box (Fig. 6(b)). All the images were taken without zooming in on the visuals. The light intensity levels were measured at various locations on the paper and mean intensity level was determined to be 3782 lx with percentage coefficient of variation of 4.29%, confirming the uniformity of lighting conditions. The acquired digital images were transferred to a computer system for further analysis and interpretation.

Image analysis plays a significant role in the detection of heavy metal ions qualitatively and quantitatively. It could be performed using various platforms such as ImageJ, Adobe Photoshop, GIMP, etc. In this work, ImageJ, an open-source image processing program was used to analyze images (https://imagej.nih.gov/ij/index.html). A sequence of steps was performed for the image analysis. Firstly, the picture was loaded on the ImageJ platform. The area of interest was selected using the shape selection tool. After averaging the color of the required area, Red-Green-Blue (RGB) values were recorded. The final intensity values on the transformed grayscale were obtained from individual primary color intensities by Equation (1).

(1) Intensity=255-(0.2126R+0.7152G+0.0722B)

**Fig. 7:**
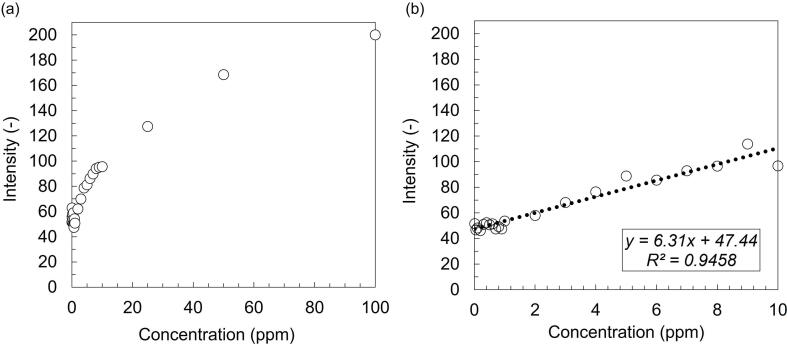
Intensity as a function of the concentration of hexavalent chromium in water samples for (a) 0 to 100 ppm concentration range, and (b) 0 to 10 ppm concentration range. The open circle symbol represents experimental data in (a) and (b) and the dotted line represents trendline fitted using linear regression analysis in (b).

The experiments were performed for water samples containing 0 to 100 ppm concentration of hexavalent chromium. The respective color changes were visually observed as well as digitally captured. The color formation was observed to have higher intensity with increased chromium concentration as shown in Fig. 6(c). The intensity was plotted as a function of concentration for the entire chromium concentration range as shown in Fig. 7(a). The nonlinearity of the plot over a wide range of concentration was attributed to surface saturation of the test zone at high chromium concentration, leading to flattening of the plot. Such nonlinear behavior is in agreement with colorimetric studies reported in literature [9].

The values followed a linear trend in the chromium concentration range of 0 to 10 ppm. A quantitative correlation was developed between intensity and concentration in 0–10 ppm concentration range using Microsoft Excel spreadsheet software. The intensity (y) varied linearly with respect to concentration (x) according to the equation y = 6.31x + 47.44 as shown in Fig. 7(b). The goodness of fit was determined using the coefficient of determination $\left(R^{2}\right)$. A high value of $R^{2}$ of 0.9458 suggested that the data was well-represented by the mathematical expression. The quantified relation could be reliably used for predicting the concentration of unknown chromium samples tested using the same methodology.

**Fig. 8:**
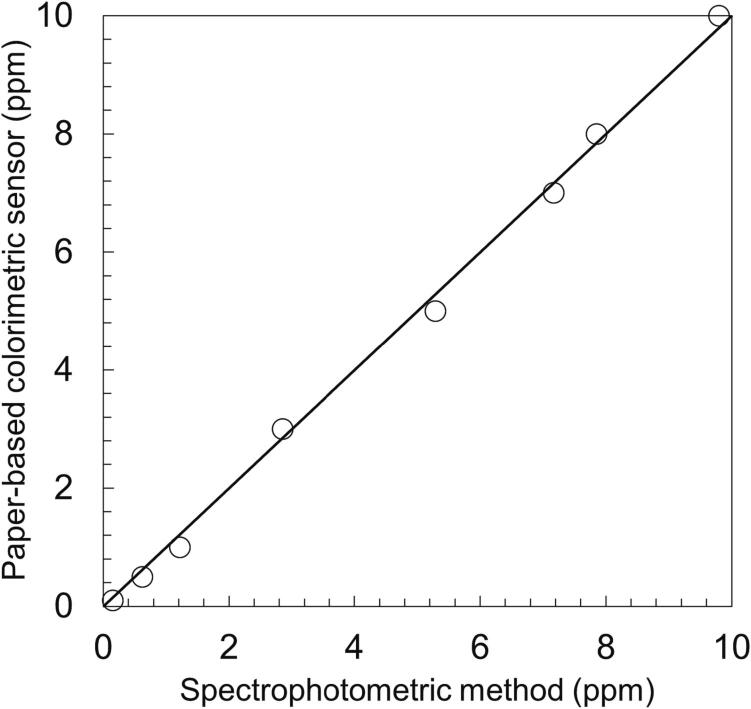
Comparison of hexavalent chromium concentration values (in ppm) determined using paper-based colorimetric sensor and spectrophotometer.

The results of hexavalent chromium analyzed by the paper-based colorimetric method were compared with standard ultraviolet–visible spectrophotometric method. Fig. 8 shows a comparative plot of chromium concentration measured by both methods, with a diagonal line denoting the line of equality. The location of data points near to the diagonal line suggested a good agreement in the concentration measurements by both analytical methods. The observations confirmed the accuracy of the proposed paper-based colorimetric sensor. The simple and frugal approach for chromium estimation demonstrated on the paper-based analytical device is especially useful in locations that do not have easy access to sophisticated atomic absorption spectroscopy or ultraviolet–visible spectrophotometry.

## 5 Conclusion

The protocol for developing a microfluidic paper-based device coupled with an imaging box suitable for digital colorimetric detection of analytes is presented in this work. The device can be easily fabricated using economic and readily available off-the-shelf components. The users can employ the device to perform multiplexed sensing and follow a similar experimental approach for parallel detection of multiple analytes. The design can also be modified to suit specific requirements. In such a case, the standard biopsy punch can be substituted by the customized cutting tools to fabricate the designed device using a similar methodology. The cost of device fabrication and reagent volume requirements are relatively very low, bringing down the cost of the analytical assay. Using paper as a substrate makes the approach more environmentally friendly and sustainable. Although the fabrication cost of the imaging box is relatively higher compared to that of paper-based devices, it is a single-time cost. It can be used even for other applications that need constant illumination conditions. The motivation of the work lies in developing a system for detecting contaminants in resource-limited settings such as remote areas, rural locations, moderately and poorly equipped nations, and regions. Thus, a frugal approach is adopted to provide a simple setup for analytical detection that could aid in rapid and affordable detection. Further capabilities can be introduced in the imaging box to provide controlled illumination, temperature, humidity, universal window for image capturing, and other adjustable features to meet user requirements. The hardware and methodology demonstrated here would find useful applications in the field of environment, food quality, and healthcare.

## Declaration of Competing Interest

The authors declare that they have no known competing financial interests or personal relationships that could have appeared to influence the work reported in this paper.
